# Sex-dependent variability of isoniazid and rifampicin serum levels in patients with tuberculosis

**DOI:** 10.1007/s15010-024-02424-5

**Published:** 2024-11-12

**Authors:** Raja Idris, Alexander Z. Dayani, Ana M. Groh, André Mohr, Julia Koepsell, Ann-Sophie Zielbauer, Eva Herrmann, Maria J. G. T. Vehreschild, Thomas A. Wichelhaus, Nils Wetzstein

**Affiliations:** 1 Department of Internal Medicine, Infectious Diseases, University Hospital, Goethe University Frankfurt, Theodor-Stern-Kai 7, 60590 Frankfurt am Main, Germany; 2 Mycobacterial Infection Research Unit (MIRU), Goethe University Frankfurt, Frankfurt am Main, Germany; 3 Hospital Pharmacy, University Hospital, Goethe University Frankfurt, Frankfurt am Main, Germany; 4 Institute of Biostatistics and Mathematical Modeling, Goethe University Frankfurt, Frankfurt am Main, Germany; 5 Institute of Medical Microbiology and Infection Control, University Hospital, Goethe University Frankfurt, Frankfurt am Main, Germany; 6 Molecular and Experimental Mycobacteriology, Research Center Borstel, Borstel, Germany

**Keywords:** Tuberculosis, TDM, Drug levels, Rifampicin, Isoniazid, Sex

## Abstract

**Introduction:**

Drug-sensitive TB (DS-TB) is treated with isoniazid, rifampicin, ethambutol, and pyrazinamide. Factors like fast-metabolizing enzymes, malabsorption, and drug interactions can influence serum drug levels. Current TB treatment guidelines recommend weight-adapted dosing without considering sex differences. This study examines drug levels of isoniazid and rifampicin in TB patients treated between 2019 and 2023 at our center focusing on sex-specific aspects.

**Methods:**

Patients diagnosed with TB and available serum levels of isoniazid or rifampicin between 2019 and 2023 were retrospectively identified. Serum levels were measured using liquid chromatography–mass spectrometry and high-performance liquid chromatography. Patients were stratified by sex and a linear regression mixed effect model was used to assess predictors for different serum levels.

**Results:**

The study included 281 single therapeutic drug monitoring (TDM) measurements from 59 patients (28 women, 47.5%). For isoniazid, no sex-specific differences in serum drug levels were identified. On the other hand, female sex was a significant predictor of higher rifampicin plasma levels (coefficient 4.16, 95% CI 0.74–7.59, *p* = 0.009). Only 38.2% of rifampicin serum level measurements in male patients were within target range, the majority (40/68, 58.8%) were below range and only 2 (2.9%) TDM-levels were above range. Women displayed higher overall rifampicin serum levels than men (median 13.7 mg/l vs. 7.1 mg/l, *p* = 0.04), although weight adjusted doses were not significantly different (median 10.0 mg/kg vs. 9.8 mg/kg *p* = 0.56). Adverse effects were noted in 42.9% (42/98) of measurements in women and 29.5% (54/183) of measurements in men (*p* = 0.03).

**Discussion:**

Rifampicin levels were significantly lower in men compared to women, despite weight-adjusted dosing. Clinicians should consider TDM and potential sex differences when treating patients with TB.

**Supplementary Information:**

The online version contains supplementary material available at 10.1007/s15010-024-02424-5.

## Introduction

Tuberculosis (TB) is caused by bacteria of the *Mycobacterium tuberculosis* complex (Mtbc) and is, according to the WHO (World Health Organization), the second deadliest infectious disease caused by a single pathogen after SARS-CoV 2 [1]. In 2022, 7.5 million reported cases and an estimated 1.3 million deaths were announced by the WHO, which is the highest number of new cases since the beginning of the global TB monitoring program in 1995 [2]. It is estimated that the disruptions in case identification, screening and treatment due to the COVID-19 pandemic led to 500,000 excess deaths in the years 2020–2022 [2]. Therefore, TB continues to be a major burden on global health.

Drug-sensitive TB (DS-TB) is treated with a standard regimen consisting of isoniazid, rifampicin, ethambutol and pyrazinamide [3]. Doses are generally weight-adapted, in order to achieve sufficient plasma levels [4]. However, patients may be under- or overdosed due to several factors, such as genetic polymorphisms of drug metabolizing enzymes (e.g. fast and slow metabolizers) as well as malabsorption, or interactions with drug- and food, respectively. Standard doses of many TB therapies are of historic origin and were chosen with regard to effectiveness, adverse effects and cost without greater evidence based on dose-finding studies [5]. Especially for rifampicin, dose adjustments are frequently necessary and the standard doses of 10 mg/kg body weight with a maximum of 600 mg/d might often not be sufficient [6]. To avoid incorrect dosing, many clinicians turn to therapeutic drug monitoring (TDM), especially in cases where an insufficient therapeutic response is observed.

TDM is a way to utilize plasma drug concentrations to make informed decisions about drug dosing [7]. In TB treatment, TDM is not yet part of the standard of care and is primarily available in some high- and upper-middle-income countries [8]. WHO recommends TDM in specific treatment situations, such as the therapy of multidrug resistant tuberculosis (MDR-TB) [9]. Until now it is not clear, if low plasma drug levels correlate with adverse treatment outcomes [10].

In recent years, sex has become widely recognized as an important factor contributing to the susceptibility and immune response to infectious diseases [11, 12]. For instance, TB prevalence is lower among women [13], who are also more likely to present with extrapulmonary disease [14] and seem to experience side effects from tuberculostatic therapy more often [15]. These epidemiological differences do not appear to exist in children before the onset of puberty [16]. Current guidelines for the treatment of TB recommend weight-adapted dosing of the standard tuberculostatic drugs [17]. However, differences in dosing regarding the patient’s sex are generally not recommended [3, 16].

In this study, we provide detailed drug level measurement data of isoniazid and rifampicin in a cohort of adult patients with TB treated at our center in Frankfurt am Main, Germany, between 2019 and 2023 and examine influential factors on these levels including sex specific aspects.

## Methods

### Included patients

All adult patients (18 years or older) with a microbiological or clinical diagnosis of TB and at least one serum level for isoniazid or rifampicin between 2019 and 2023 were retrospectively identified. Patients with insufficient clinical data were excluded from the analysis (Figure S1). We recorded clinical characteristics, such as comorbidities (including diabetes, HIV, chronic kidney disease, chronic vascular disease, smoking habits, malignancy, pregnancy, and immunosuppression), the clinical manifestation of TB, geographical origin, and weight by retrospective chart review from our local patient data management system (ORBIS, Dedalus Healthcare Systems Group, Bonn, Germany). Cases with confirmed exclusive lung involvement were classified as pulmonary TB, those with exclusively extrapulmonary manifestations as extrapulmonary TB, and cases with both pulmonary and extrapulmonary involvement as disseminated TB. Antimycobacterial therapy, side effects, treatment outcome, microbiological characteristics, such as the isolated species of the *M. tuberculosis* complex and drug resistance patterns were recorded, as well. The tuberculosis medication dosage was determined according to the German guidelines [17], prescribing a rifampicin dose of 10 mg/kg body weight (range 8–12 mg/kg) and an isoniazid dose of 5 mg/kg body weight (range 4–6 mg/kg) [17]. For statistical analyses the patients were stratified by sex (defined as the biological sex at birth). This study was approved by the local ethics committee (file number 2023 − 1570).

### Therapeutic drug monitoring of isoniazid and rifampicin

Serum levels of isoniazid and rifampicin were assessed as part of the clinical routine in the infectious disease outpatient clinic and ward of the University Hospital in Frankfurt, Germany. The measurements were performed at different time points (either 0, 1, 2, 3, 4, and > 4 h) after drug intake. Concentrations were measured at the Bioscientia laboratory, Bremen, Germany. Rifampicin levels were measured by liquid chromatography–mass spectrometry (LC–MS) and isoniazid levels were determined by high-performance liquid chromatography (HPLC).

Reference ranges for measurements after intake were defined as 3 to 6 mg/l for isoniazid [18] and 8 to 24 mg/l for rifampicin [19]. The reason (routine, adverse effects, insufficient response, plasma Ievels too high, plasma Ievels too Iow) for each serum level measurement, as well as its consequence (no change, timely retesting, increase of dose, decrease of dose, increase of duration of therapy, change of route of administration, change of substance) were recorded. In addition, clinical characteristics, such as response to therapy and side effect were recorded for each point of measurement.

### Statistical analysis

All statistical analyses were performed in R v. 4.3.1 („Beagle Scouts“) [20] with packages of the *tidyverse* [21]. Patients were stratified by sex defined as biological sex recorded in our hospital databases. Normally distributed numeric data is presented as mean with standard deviation, non-normally distributed data as median with interquartile range and range. Normality was tested for using the Shapiro-Wilk-Test. Categorical data is presented as numerator with denominator and percentages. A linear regression mixed effect model accounting for the patient as permanent factor influencing the drug level was performed with the *finalfit* and the *lme4* packages within *R* [22]. For this, only peak serum levels (at hours 1 or 2) and a single measurement per patient were used. Results are depicted as coefficients with 95% confidence intervals (95% CI) and p-values. For all statistical tests a significance level of alpha = 0.05 was used.

## Results

### Baseline characteristics

**Fig. 1:**
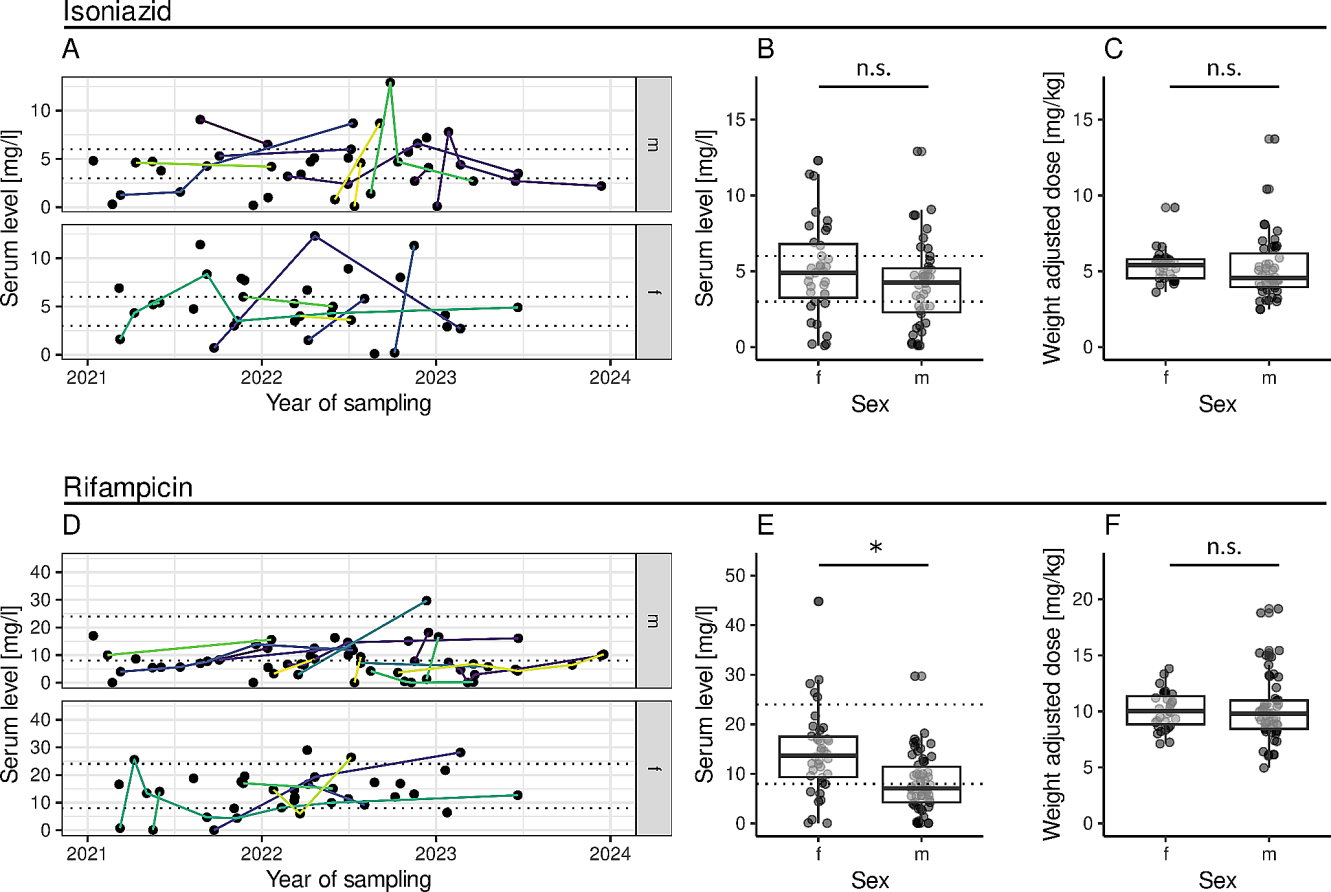
Serum levels for isoniazid and rifampicin over time (**A**, **D**), stratified by gender (**B**, **E**) and weight adjusted dose stratified by gender (**C**, **F**). Dotted lines in figure panel **A**, **B**, **D**, and **E** represent target ranges of isoniazid and rifampicin respectively. Repeat measurements from the same patients are connected by continuous lines in different colors (**A**, **D**). f = female; m = male; n.s. = not significant; significant differences are depicted by an asterisk

A total of 59 patients, 28 women and 31 men, and 281 single TDM measurements were included in the study (Table 1; Fig. 1A and D). The mean age was 36.8 years. Most patients suffered from an infection with *Mycobacterium tuberculosis*, but two patients were found to be infected with *Mycobacterium africanum* and one patient with *Mycobacterium bovis* (Table 1). There was a high rate of extrapulmonary tuberculosis (EPTB) among women (17/28, 60.7%). Only 7.1% (2/28) of women had isolated pulmonary tuberculosis and 32.1% (9/28) suffered from disseminated disease. Meanwhile, 22.6% (7/31) of the male subjects were found to have isolated pulmonary tuberculosis, 19.4% (6/31) had extrapulmonary TB and 58.1% (18/31) had disseminated TB (Fisher exact test for extrapulmonary TB, *p* = 0.003). The most frequently affected organs were the lungs (50.8%), the lymph nodes (42.4%) and the bones (25.4%). Around 85% (44/52) of the cases were drug-sensitive TB (DS-TB) without any form of resistance, in 13.5% (7/52) monoresistance was found and one case with polyresistance (isoniazid and streptomycin) was included (Table S1).

**Table 1:** Baseline characteristics of included patients

	All (*n* = 59)	Women (*n* = 28)	Men (*n* = 31)
	n (%)	n (%)	n (%)
Age mean (range)	36.8 (18–76)	34.8 (18–61)	38.6 (18–76)
Weight mean (range) [kg]	68.2 (32.8–121)	50.6 (35.3–84.4)	73.4 (32.8–121)
Comorbidities			
HIV	2 (3.4)	1 (3.6)	1 (3.2)
Immunosuppressed (excluding HIV)	3 (5.1)	2 (7.1)	1 (3.2)
Diabetes	4 (6.8)	3 (10.7)	1 (3.2)
Malignancy	2 (3.4)	1 (3.6)	1 (3.2)
CVD	6 (10.2)	4 (14.3)	2 (6.5)
Smoker	15 (25.4)	3 (10.7)	12 (38.7)
CKD	4 (6.8)	2 (7.1)	2 (6.5)
Pregnancy	n.a.	1 (3.6)	n.a.
WHO region			
African Region	12 (20.3)	6 (21.4)	6 (19.4)
Eastern Mediterranean Region	17 (28.8)	6 (21.4)	11 (35.5)
European Region	10 (16.9)	2 (7.1)	8 (25.8)
Region of the Americas	0 (0.0)	0 (0.0)	0 (0.0)
South-East Asia Region	12 (20.3)	7 (25.0)	5 (16.1)
Western Pacific Region	6 (10.2)	5 (17.9)	1 (3.2)
n.a.	0 (0.0)	2 (7.1)	0 (0.0)
Clinical manifestation of TB			
Isolated pulmonary	9 (15.3)	2 (7.1)	7 (22.6)
Extrapulmonary	23 (39.0)	17 (60.7)	6 (19.4)
Disseminated	27 (45.8)	9 (32.1)	18 (58.1)
Specific organ manifestations			
Lung	30 (50.8)	9 (32.1)	21 (67.7)
Pleura	5 (8.5)	1 (3.6)	4 (12.9)
Lymph node	25 (42.4)	13 (46.4)	12 (38.7)
Abdominal	13 (22.0)	4 (14.3)	9 (29.0)
Bone	15 (25.4)	7 (25.0)	8 (25.8)
Urogenital	4 (6.8)	0 (0.0)	4 (12.9)
CNS	7 (11.9)	3 (10.7)	4 (12.9)
Spine	11 (18.6)	5 (17.9)	6 (19.4)
Other	5 (8.5)	1 (3.6)	4 (12.9)
Species			
*Mycobacterium tuberculosis*	49 (83.1)	22 (78.6)	27 (87.1)
*Mycobacterium tuberculosis* complex (not further specified)	7 (11.9)	5 (17.9)	2 (6.5)
*Mycobacterium africanum*	2 (3.4)	1 (3.6)	1 (3.2)
*Mycobacterium bovis*	1 (1.7)	0 (0.0)	1 (3.2)

kg = kilogram; HIV = Humane Immunodeficiency Virus; CVD = cardiovascular disease; CKD = chronic kidney disease; CNS = central nervous system; WHO = World Health Organization; n.a. = no answer

### Drug levels at different time points

**Fig. 2:**
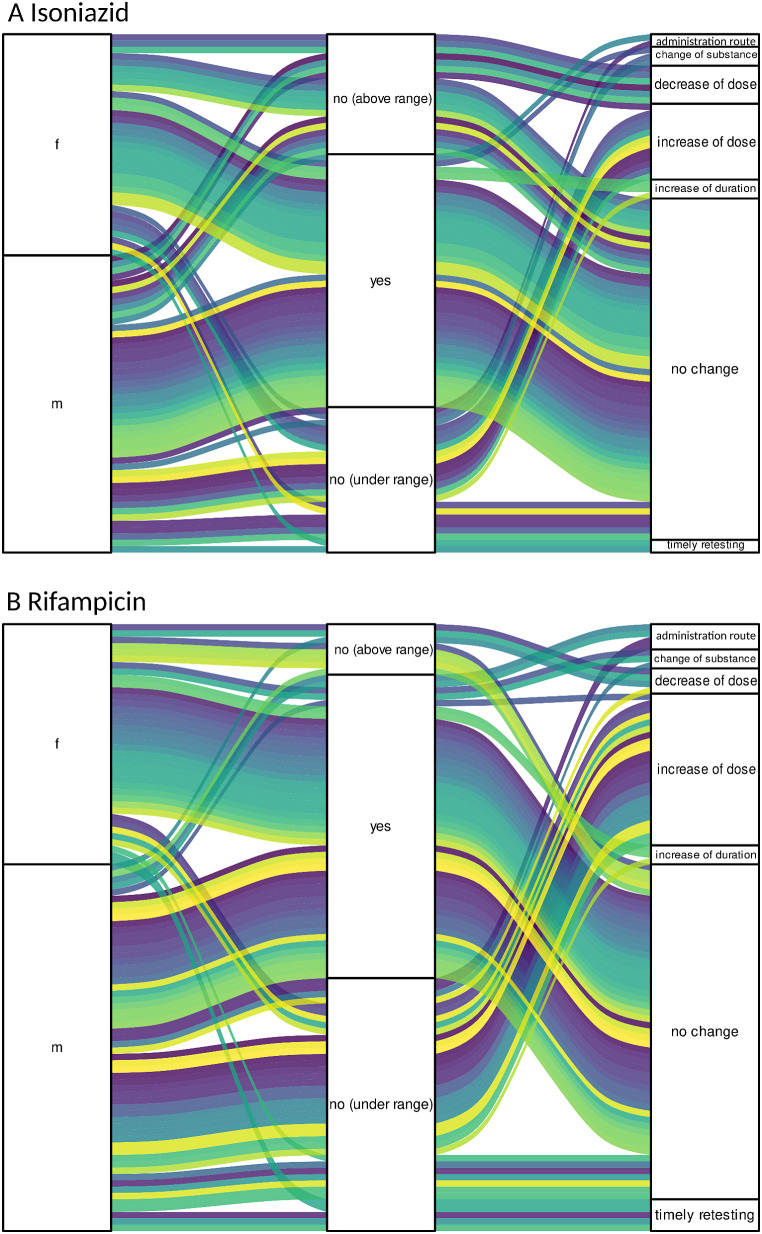
Individual trajectories of drug levels in female and male patients (first column), their results (middle column) and consequence (right column) for isoniazid serum levels (**A**) and rifampicin levels (**B**). Colours indicate individual patients. f = female; m = male

We included 281 single TDM measurements (Table 2; Fig. 1A and D). There was no significant difference between measurements at one hour after intake and two hours after intake for both antimycobacterial agents (Figure S2). Individual trajectories of drug levels in female and male patients, their results and consequences are depicted in Fig. 2 and supplemental Figure S3. Routine checks of serum concentrations were the most common reason for TDM (161/281, 57.3%), followed by an insufficient clinical response (55/281, 19.6%), insufficient plasma levels on last measurement (49/281, 17.4%), increased plasma levels on last measurement (6/281, 2.1%) and adverse effects (10/281, 3.6%) (Figure S3). Adverse effects of the antituberculous medication were noted at the time of measurements in 42.9% (42/98) of measurements in women and 29.5% (54/183) of measurements in men (*p* = 0.03). In women, the most common side effects were gastrointestinal in nature (9/98, 9.2%), closely followed by increases in liver enzymes (8/98, 8.2%) (Table S2). Less gastrointestinal adverse effects were observed in men at the time of TDM (12/183, 6.6% of measurements) an increase in liver enzymes was only noted in 1.1% (2/183).

### Isoniazid

The mean dose of Isoniazid was 317 mg (range 200-600 mg) with a slightly lower dose in women (284 mg, range 200–600 mg) than in men (338 mg, range 200–600) (Table 2). Overall, isoniazid levels were not significantly different between female and male patients (median 4.9 mg/ml, IQR 3.3–6.8 vs. 4.3 mg/ml, IQR 2.3–5.2, *p* = 0.16) (Fig. 1B), as well as weight adapted doses (5.4 mg/kg, IQR 4.5–5.8 vs. 4.5 mg/kg, IQR 4.0–6.2, *p* = 0.11) (Fig. 1C). However, women were more likely to have a peak serum level within the target range (3 to 6 mg/l, 18/35, 51.4%) or above (9/35, 25.7%), while only 22.9% (8/35) of measurements were below (Table 2; Fig. 2A). In the men’s group, only 44.0% (22/50) of peak serum levels were within and 20.0% (10/50) above the target range, while 36.0% (18/50) were below. Following the majority of TDM measurements (63.1%) in both women and men no change to the drug regimen or the dosages was deemed necessary (Fig. 2A).

**Table 2:** Overview of dosing and serum levels of isoniazid and rifampicin

	All (*n* = 59)	Women (*n* = 28)	Men (*n* = 31)
	n/N (%)	n/N (%)	n/N (%)
Number of all TDM measurements	281	98	183
peak level measurements	191/281 (68.0)	75/98 (76.5)	116/183 (63.4)
through level measurements	51/281 (18.1)	14/98 (14.3)	37/183 (20.2)
type of measurements unknown	39/281 (13.9)	9/98 (9.2)	30/183 (16.4)
Isoniazid			
number of TDM measurements	124/281 (44.1)	47/98 (48.0)	77/183 (42.1)
dose mean (range) [mg]	317.3 (200–600)	284 (200–600)	337.7 (200–600)
dose median per bodyweight (IQR)[mg/kg]	4.9 (4.2–5.8)	5.4 (4.5–5.8)	4.5 (4.0-6.2)
dose category			
200 mg	8/124 (6.5)	5/47 (10.6)	3/77 (3.9)
250 mg	19/124 (15.3)	14/47 (29.8)	5/77 (6.5)
300 mg	71/124 (57.3)	26/47 (55.3)	45/77 (58.4)
350 mg	6/124 (4.8)	0/47 (0.0)	6/77 (7.8)
400 mg	6/124 (4.8)	0/47 (0.0)	6/77 (7.8)
450 mg	8/124 (6.5)	1/47 (2.1)	7/77 (9.1)
600 mg	6/124 (4.8)	1/47 (2.1)	5/77 (6.5)
peak level (*n* = 85)			
above range	19/85 (22.4)	9/35 (25.7)	10/50 (20.0)
below range	26/85 (30.6)	8/35 (22.9)	18/50 (36.0)
within target range	40/85 (47.1)	18/35 (51.4)	22/50 (44.0)
Rifampicin			
number of TDM measurements	157/281 (55.9)	51/98 (52.0)	106/183 (57.9)
dose mean (range) [mg]	649.7 (450–1200)	567.6 (450–900)	689.2 (450–1200)
dose median per bodyweight (IQR)[mg/kg]	9.8 (8.6–11.1)	10.0 (8.8–11.4)	9.8 (8.4–11.0)
dose category			
450 mg	18/157 (11.5)	16/51 (31.4)	2/106 (1.9)
600 mg	96/157 (61.1)	32/51 (62.7)	64/106 (60.4)
750 mg	18/157 (11.5)	1/51 (2.0)	17/106 (16.0)
900 mg	24/157 (15.3)	2/51 (3.9)	22/106 (20.8)
1200 mg	1/157 (0.6)	0/51 (0.0)	1/106 (0.9)
peak level (*n* = 106)			
above range	8/106 (7.5)	6/40 (15.0)	2/66 (3.0)
below range	48/106 (45.3)	8/40 (20.0)	40/66 (60.6)
within target range	52/106 (49.1)	26/40 (65.0)	26/66 (39.4)

### Rifampicin

Mean dose of Rifampicin in women was 568 mg (range 450–900 mg) and in men 689 mg (450–1200 mg) (Table 2). Women displayed higher overall rifampicin serum levels than men (median 13.7 mg/l, IQR 9.3–17.5 vs. median 7.1 mg/l, IQR 4.3–11.4, Fig. 1E), although weight adjusted doses were equal (median 10.0 mg/kg with IQR 8.8–11.4 vs. median 9.8 mg/kg with IQR 8.4–11.0, *p* = 0.56, Fig. 1F). Women were also more likely to have a peak serum level within or above the target range of 8 to 24 mg/l (32/40, 80.0%), while 20% (8/40) were below (Table 2; Fig. 2B). Only 38.2% (26/68) of measurements in the men’s group were within target range, the majority (40/68, 58.8%) were below range and only 2 out of 68 (2.9%) TDM-levels were above range. An increase in rifampicin dosage was documented as a result of TDM in 29.7% of measurements in men, the route of administration was changed in 7.7% of cases, the drug regimen was altered in 2.2%, and timely retesting was performed in 11.0% (Fig. 2B). In contrast, after the majority of measurements in women (68.1%) no change to the drug regimen or the dosages was deemed necessary (Fig. 2B).

### Predictors of drug levels

**Fig. 3:**
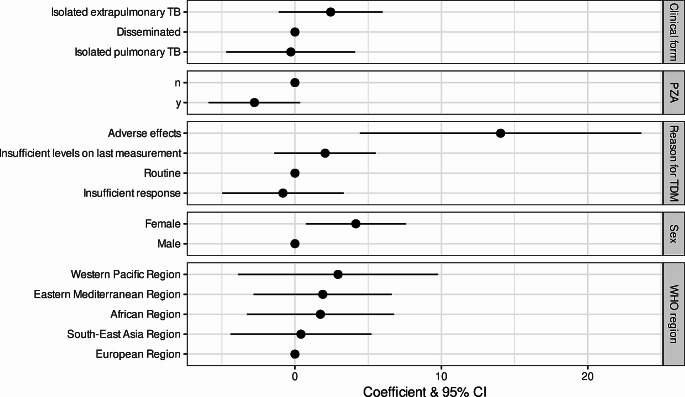
Coefficients and 95% confidence intervals for the prediction of rifampicin serum levels in the multivariable model. 95% CI = 95% confidence interval; n = no, y = yes, PZA = pyrazinamide; TDM = therapeutic drug monitoring; WHO = World Health Organization; TB = tuberculosis

For isoniazid, the administered dose (coefficient 0.01 95% CI 0.00-0.02, *p* = 0.003) and a co-administration of rifampicin (coefficient-7.18 95% CI -12.45-1.91, *p* = 0.004) were significantly associated with higher or lower plasma levels of the drug in the multivariable analysis, respectively (Table S3). For rifampicin, the patient’s sex as well as the adverse effects as reason for the drug level testing were the strongest predictors for higher plasma levels of rifampicin (coefficient for sex 4.16 95% CI 0.74–7.59, *p* = 0.009, and for adverse effects 14.05 95% CI 4.44–23.66, *p* = 0.002) (Table S4, Fig. 3).

## Discussion

In this study, we found that men with tuberculosis exhibit lower rifampicin serum levels than women despite adequate weight adjusted dosing. More than half of rifampicin serum levels in male patients were below the target range, while this was only the case in a minority of measurement in women. We therefore provide real-world evidence that male patients might benefit from higher rifampicin doses and that TDM in this patient group seems to be important to determine, monitor and therefore achieve sufficient plasma levels of rifampicin. Differential sex-specific aspects of antimicrobial therapy are not widely discussed, although other studies have shown similar results [23–25]. The reasons behind lower rifampicin levels in men are unclear, McIlleron et al. hypothesized that this may be attributed to the higher lean body mass to total weight ratios in men [23]. In contrast to female sex, factors such as alcohol use, age, smoking, and duration of drug therapy were found to be unsuitable predictors of rifampicin exposure in a study by Sileshi et al. involving 119 tuberculosis patients in Ethiopia [24]. They also found that certain genetic variations in enzymes and transporter proteins involved in rifampicin metabolism correlated with lower rifampicin levels; however, the study did not mention any sex-specific differences in the occurrence of these variations [24]. Generally, a higher activity level of cytochrome p450 isoenzymes in men and longer retention time of gastric contents in women are supposed to contribute to sex-specific differences in pharmacokinetics [26–27].

In line with the finding of lower (and even subtherapeutic) rifampicin levels in men is the fact that only a small fraction of male patients experienced an increase of liver enzymes in this cohort. Overall, we observed a relatively high rate of self-reported adverse effects in 42.9% of measurements in women and a lower rate of 29.5% in men. Other studies have also found that side effects due to tuberculostatic therapy are more frequent in female patients [28, 29].

The real-world impact of our findings on treatment success rates is unclear, but a recent systematic review showed that in Europe the case fatality rate is 30–50% higher in males than in females [30]. Different studies have also demonstrated better treatment response in women [31] and higher mortality rates in males with tuberculosis [32]. Insufficient drug levels have been associated with unfavorable treatment outcomes in other investigations [33–35]. Further, inadequate isoniazid [35] and rifamycin [36] levels were shown to be associated with acquired drug resistance [36]. If these associations are confirmed, higher rifampicin doses could benefit not only individual patients, but also global health efforts in combating the rise of MDR TB. The current dosing recommendation for rifampicin has also been contested by others as higher doses seem to be safe and promise higher efficacy [6, 37]. These aspects are, however, not part of current treatment guidelines [3, 4, 17, 38]. This underlines the necessity of an individualized therapeutic approach even in standard TB therapy that takes the patient’s sex into account.

This study has several limitations: despite including 59 patients with 281 TDM measurements, the sample size is limited. As TDM levels were limited to single time point measurements, delayed absorption or total drug exposure might not have been entirely accounted for. Additionally, TDM levels were assessed retrospectively, without a predefined study protocol. While most drug levels were obtained as part of routine care, selection bias cannot be ruled out, such as including more patients with insufficient treatment response or excluding male patients with elevated liver enzymes at therapy onset. Also, long-term observation data is not yet available, preventing us from linking these findings to potential therapeutic failures or higher TB relapse rates.

## Conclusion

In this study, rifampicin levels in men were significantly lower than in women despite weight-adjusted dosing. Clinicians should be aware of sex-specific pharmacokinetic differences in TB treatment and the performance of TDM even for standard therapy merits further investigations. These results could also point to the need of a higher rifampicin doses when treating men for tuberculosis.

## Electronic supplementary material

Below is the link to the electronic supplementary material.

Supplementary Material 1

## Data Availability

Data is provided within the manuscript or supplementary information files.

## Abbreviations

DS-TB: Drug sensitive tuberculosis
EPTB: Extrapulmonary tuberculosis
HIV: Human immunodeficiency virus immunodeficiency virus
HPLC: High-performance liquid chromatography (HPLC)
LC-MS: Liquid chromatography– mass spectrometry (LC–MS)
MDR-TB: Multidrug resistant tuberculosis
Mtbc: Mycobacterium tuberculosis complex
TB: Tuberculosis
TDM: Therapeutic drug monitoring
WHO: World Health Organization
