# Supplementary material for: Sex-dependent variability of isoniazid and rifampicin serum levels in patients with tuberculosis
Source: Infection. 2024 Nov 12;53(3):1051–60. doi: 10.1007/s15010-024-02424-5 (PMC12137477; doi:10.1007/s15010-024-02424-5)
Supplement: Supplementary file 1 — Supplementary Material 1 [file 15010_2024_2424_MOESM1_ESM.docx]

**Supplemental tables:**

Table S1: Drug susceptibility patterns and administered antimycobacterial agents in included patients.

|  | All (n=59) | Women (n=28) | Men (n=31) |
| --- | --- | --- | --- |
|  | n/N (%) | n/N (%) | n/N (%) |
| Susceptibility pattern |  |  |  |
| Drug-susceptible TB | 44/52 (84.6%) | 20/23 (87.0%) | 24/29 (82,8%) |
| Monoresistance | 7/52 (13.5%) | 3/23 (13.0%) | 4/29 (13.8%) |
| Isoniazid | 3/52 (5.8%) | 2/23 (8.7%) | 1/29 (3.4%) |
| Pyrazinamide | 1/52 (1.9%) | 0/23 (0.0%) | 1/29 (3.4%) |
| Streptomycin | 4/52 (7.7%) | 1/23 (4.3%) | 2/29 (6.9%) |
| Polyresistance (Isoniazid + Streptomycin) | 1/52 (1.9%) | 0/23 (0.0%) | 1/29 (3.4%) |
| n/a | 7/59 (11.9%) | 5/28 (17.9%) | 2/31 (6.5%) |
| Antimycobacterial treatment |  |  |  |
| Isoniazid | 57/59 (96.6%) | 27/28 (96.4%) | 30/31 (96.8%) |
| Rifampicin | 59/59 (100.0%) | 28/28 (100.0%) | 31/31 (100.0%) |
| Ethambutol | 54/59 (91.5%) | 25/28 (89.3%) | 29/31 (93.5%) |
| Pyrazinamide | 58/59 (98.3%) | 28/28 (100.0%) | 30/31 (96.8%) |
| Fluoroquinolone |  |  |  |
| Levofloxacin | 22/59 (37.3%) | 8/28 (28.6%) | 14/31 (45.2%) |
| Ciprofloxacin | 1/59 (1.7%) | 0/28 (0.0%) | 1/31 (3.2%) |
| Moxifloxacin | 19/59 (32.2%) | 8/28 (28.6%) | 11/31 (35.5%) |
| Amikacin | 14/59 (23.7%) | 4/28 (14.3%) | 10/31 (32.3%) |
| Linezolid | 10/59 (16.9%) | 6/28 (21.4%) | 4/31 (12.9%) |
| Cycloserin/Terizidon | 1/59 (1.7%) | 1/28 (3.6%) | 0/31 (0.0%) |
| Prothionamide | 1/59 (1.7%) | 1/28 (3.6%) | 0/31 (0.0%) |
| Clofazimine | 3/59 (5.1%) | 1/28 (3.6%) | 2/31 (6.5%) |
| Bedaquiline | 2/59 (3.4%) | 1/28 (3.6%) | 1/31 (3.2%) |
| P-Aminosalicylic acid | 1/59 (1.7%) | 0/28 (0.0%) | 1/31 (3.2%) |
| ART (Emtricitabine/Tenofovir/Dolutegravir) | 2/59 (3.4%) | 1/28 (3.6%) | 1/31 (3.2%) |
| n/a = no answer; ART = antiretroviral therapy | | | |

Table S2: Observed side effects in included patients.

|  | All (n=59) | Women (n=28) | Men (n=31) |
| --- | --- | --- | --- |
|  | n/N (%) | n/N (%) | n/N (%) |
| Number of TDM measurements | 281 | 98 | 183 |
| Side effects |  |  |  |
| any side effects | 96 (34.2%) | 42 (42.9%) | 54 (29.5%) |
| elevated liver enzymes | 10 (3.6%) | 8 (8.2%) | 2 (1.1%) |
| gastrointestinal | 21 (7.5%) | 9 (9.2%) | 12 (6.6%) |
| arthralgia | 9 (3.2%) | 4 (4.1%) | 5 (2.7%) |
| cephalgia | 7 (2.5%) | 6 (6.1%) | 1 (0.5%) |
| blood count | 2 (0.7%) | 2 (2.0%) | 0 (0.0%) |
| skin reactions | 7 (2.5%) | 0 (0.0%) | 7 (3.8%) |
| neurological | 10 (3.6%) | 0 (0.0%) | 10 (5.5%) |
| visual auditory | 6 (2.1%) | 4 (4.1%) | 2 (1.1%) |
| drug fever | 0 (0.0%) | 0 (0.0%) | 0 (0.0%) |
| other side effects | 48 (17.1%) | 11 (11.2%) | 37 (20.2%) |
| TDM = therapeutic drug monitoring | | | |

Table S3: Univariable and multivariable linear regression for predictors of drug levels in isoniazid.

| Independent variables | Mean (SD) | Coefficient (univariable) | Coefficient (multilevel) |
| --- | --- | --- | --- |
| gender |  |  |  |
| m | 4.1 (2.8) | - | - |
| f | 5.1 (3.1) | 0.93 (-0.40 to 2.26, p=0.169) | - |
| migration |  |  |  |
| n | 3.7 (2.4) | - | - |
| missing | 4.3 (1.8) | 0.53 (-2.72 to 3.78, p=0.747) | - |
| y | 4.7 (3.3) | 1.00 (-2.07 to 4.08, p=0.517) | - |
| dose [200.0 - 600.0] | 4.6 (3.0) | 0.01 (0.00 to 0.02, p=0.003) | 0.01 (0.00-0.02, p=0.003) |
| side effects |  |  |  |
| n | 4.4 (3.1) | - | - |
| y | 4.9 (2.8) | 0.43 (-1.07 to 1.93, p=0.570) | - |
| weight [32.8,121.0] | 4.6 (3.0) | -0.02 (-0.06 to 0.02, p=0.344) | - |
| age [16.0,76.0] | 4.6 (3.0) | 0.01 (-0.05 to 0.07, p=0.745) | - |
| reason for TDM |  |  |  |
| insufficient response | 3.4 (3.5) | -4.33 (-10.38 to 1.71, p=0.158) | - |
| last plasma level too low | 5.9 (3.4) | -1.81 (-7.92 to 4.30, p=0.557) | - |
| routine | 4.5 (2.7) | -3.17 (-9.05 to 2.72, p=0.287) | - |
| rifampicin |  |  |  |
| n | 9.7 (4.4) | - | - |
| y | 4.4 (2.7) | -5.32 (-8.58 to -2.05, p=0.002) | -7.18 (-12.45--1.91, p=0.004) |
| pyrazinamide |  |  |  |
| n | 5.0 (2.7) | - | - |
| y | 4.0 (3.2) | -1.02 (-2.36 to 0.32, p=0.135) | - |
| ethambutol |  |  |  |
| n | 4.6 (2.9) | - | - |
| y | 4.4 (3.0) | -0.26 (-1.75 to 1.23, p=0.730) | - |
| levofloxacin |  |  |  |
| n | 4.7 (2.7) | - | - |
| y | 4.0 (3.6) | -0.68 (-2.23 to 0.87, p=0.385) | - |
| moxifloxacin |  |  |  |
| n | 4.5 (2.9) | - | - |
| y | 4.9 (5.8) | 0.32 (-3.16 to 3.81, p=0.853) | - |
| amikacin |  |  |  |
| n | 4.4 (2.8) | - | - |
| y | 8.8 (5.8) | 4.36 (0.24 to 8.48, p=0.038) | -3.95 (-10.43-2.53, p=0.116) |
| HIV |  |  |  |
| n | 4.6 (3.0) | - | - |
| y | 3.9 (2.6) | -0.74 (-3.08 to 1.60, p=0.530) | - |
| diabetes |  |  |  |
| n | 4.5 (2.9) | - | - |
| y | 5.6 (4.0) | 1.13 (-1.60 to 3.85, p=0.413) | - |
| immunosuppressed |  |  |  |
| n | 4.7 (3.0) | - | - |
| y | 3.5 (2.5) | -1.18 (-3.26 to 0.90, p=0.260) | - |
| cardiovascular disease |  |  |  |
| n | 4.5 (3.0) | - | - |
| y | 4.9 (1.6) | 0.37 (-2.66 to 3.41, p=0.807) | - |
| smoker |  |  |  |
| n | 4.8 (2.7) | - | - |
| missing | 4.1 (4.9) | -0.75 (-3.53 to 2.04, p=0.596) | - |
| y | 4.2 (3.1) | -0.65 (-2.08 to 0.79, p=0.370) | - |
| chronic kidney disease |  |  |  |
| n | 4.5 (3.0) | - | - |
| y | 5.6 (2.7) | 1.15 (-1.88 to 4.17, p=0.453) | - |
| SD = standard deviation; m = male; f = female; n = no; y = yes‍; TDM = therapeutic drug monitoring  SD – standard deviation; m – male; f – female; n – no; y – yes‍; TDM – therapeutic drug monitoring  SD – standard deviation; m – male; f – female; n – no; y – yes‍; TDM – therapeutic drug monitoring  SD – standard deviation; m – male; f – female; n – no; y – yes‍; TDM – therapeutic drug monitoring | | | |

Table S4: Univariable and multivariable linear regression for predictors of drug levels in rifampicin.

| Independent variables | Mean (SD) | Coefficient (univariable) | Coefficient (multilevel) |
| --- | --- | --- | --- |
| gender |  |  |  |
| m | 8.0 (5.8) | - | - |
| f | 14.4 (8.8) | 6.34 (3.39 to 9.29, p<0.001) | 4.16 (0.74-7.59, p=0.009) |
| migration |  |  |  |
| n | 8.8 (5.1) | - | - |
| missing | 12.3 (6.1) | 3.53 (-4.13 to 11.19, p=0.363) | - |
| y | 10.1 (8.3) | 1.34 (-5.78 to 8.47, p=0.709) | - |
| dose [450.0 - 1200.0] | 10.6 (7.7) | 0.00 (-0.01 to 0.01, p=0.957) | - |
| side effects |  |  |  |
| n | 10.0 (6.3) | - | - |
| y | 11.5 (9.9) | 1.58 (-1.74 to 4.91, p=0.347) | - |
| weight [38.9,121.0] | 10.6 (7.7) | 0.03 (-0.07 to 0.13, p=0.541) | - |
| age [17.0,75.0] | 10.6 (7.7) | -0.00 (-0.13 to 0.12, p=0.940) | - |
| reason for TDM |  |  |  |
| routine | 10.4 (8.3) | - | - |
| adverse effects | 24.6 (7.1) | 14.23 (3.38 to 25.08, p=0.011) | 14.05 (4.44-23.66, p=0.002) |
| insufficient response | 9.2 (6.1) | -1.18 (-5.66 to 3.31, p=0.604) | -0.82 (-4.98-3.33, p=0.349) |
| last plasma level too low | 10.3 (6.3) | -0.09 (-4.00 to 3.81, p=0.962) | 2.06 (-1.42-5.53, p=0.123) |
| isoniazid |  |  |  |
| n | 10.3 (12.5) | - | - |
| y | 10.6 (7.1) | 0.26 (-4.90 to 5.42, p=0.921) | - |
| pyrazinamide |  |  |  |
| n | 12.6 (8.0) | - | - |
| y | 8.1 (6.6) | -4.50 (-7.53 to -1.46, p=0.004) | -2.77 (-5.90-0.36, p=0.041) |
| ethambutol |  |  |  |
| n | 11.1 (7.0) | - | - |
| y | 9.3 (9.1) | -1.83 (-5.21 to 1.55, p=0.285) | - |
| HIV |  |  |  |
| n | 10.9 (7.9) | - | - |
| y | 8.6 (6.3) | -2.32 (-6.90 to 2.26, p=0.317) | - |
| diabetes |  |  |  |
| n | 10.2 (7.6) | - | - |
| y | 17.7 (8.4) | 7.48 (-0.25 to 15.22, p=0.058) | - |
| immunosuppressed |  |  |  |
| n | 10.9 (7.8) | - | - |
| y | 8.9 (7.2) | -2.01 (-6.33 to 2.31, p=0.358) | - |
| cardiovascular disease |  |  |  |
| n | 10.3 (7.4) | - | - |
| y | 13.7 (10.9) | 3.42 (-2.24 to 9.08, p=0.233) | - |
| smoker |  |  |  |
| n | 11.7 (8.0) | - | - |
| Missing | 11.4 (12.3) | -0.34 (-7.43 to 6.75, p=0.925) | - |
| y | 8.5 (6.2) | -3.27 (-6.56 to 0.02, p=0.052) | - |
| chronic kidney disease |  |  |  |
| n | 10.6 (7.8) | - | - |
| y | 9.9 (6.5) | -0.69 (-7.20 to 5.82, p=0.834) | - |
| no malignancy | 10.7 (7.7) | - | - |
| TB type |  |  |  |
| disseminated | 9.8 (6.6) | - | - |
| isolated extrapulmonary | 14.5 (9.0) | 4.65 (1.25 to 8.06, p=0.008) | 2.44 (-1.11-5.99, p=0.089) |
| isolated pulmonary | 6.4 (5.9) | -3.47 (-7.44 to 0.49, p=0.085) | -0.28 (-4.69-4.12, p=0.450) |
| WHO region |  |  |  |
| European Region | 6.3 (4.7) | - | - |
| African Region | 12.4 (7.9) | 6.11 (1.38 to 10.84, p=0.012) | 1.74 (-3.29-6.77, p=0.249) |
| Eastern Mediterranean Region | 10.8 (7.0) | 4.45 (0.09 to 8.80, p=0.046) | 1.90 (-2.83-6.62, p=0.216) |
| Missing | 12.7 (2.7) | 6.39 (-4.51 to 17.28, p=0.247) | 0.29 (-10.27-10.84, p=0.479) |
| South-East Asia Region | 11.4 (7.8) | 5.05 (0.74 to 9.36, p=0.022) | 0.41 (-4.40-5.22, p=0.434) |
| Western Pacific Region | 15.9 (13.1) | 9.60 (3.22 to 15.98, p=0.004) | 2.94 (-3.90-9.77, p=0.200) |
| SD = standard deviation; m = male; f = female; n = no; y = yes‍; TDM = therapeutic drug monitoring; TB = tuberculosis | | | |

**Supplemental figures:**


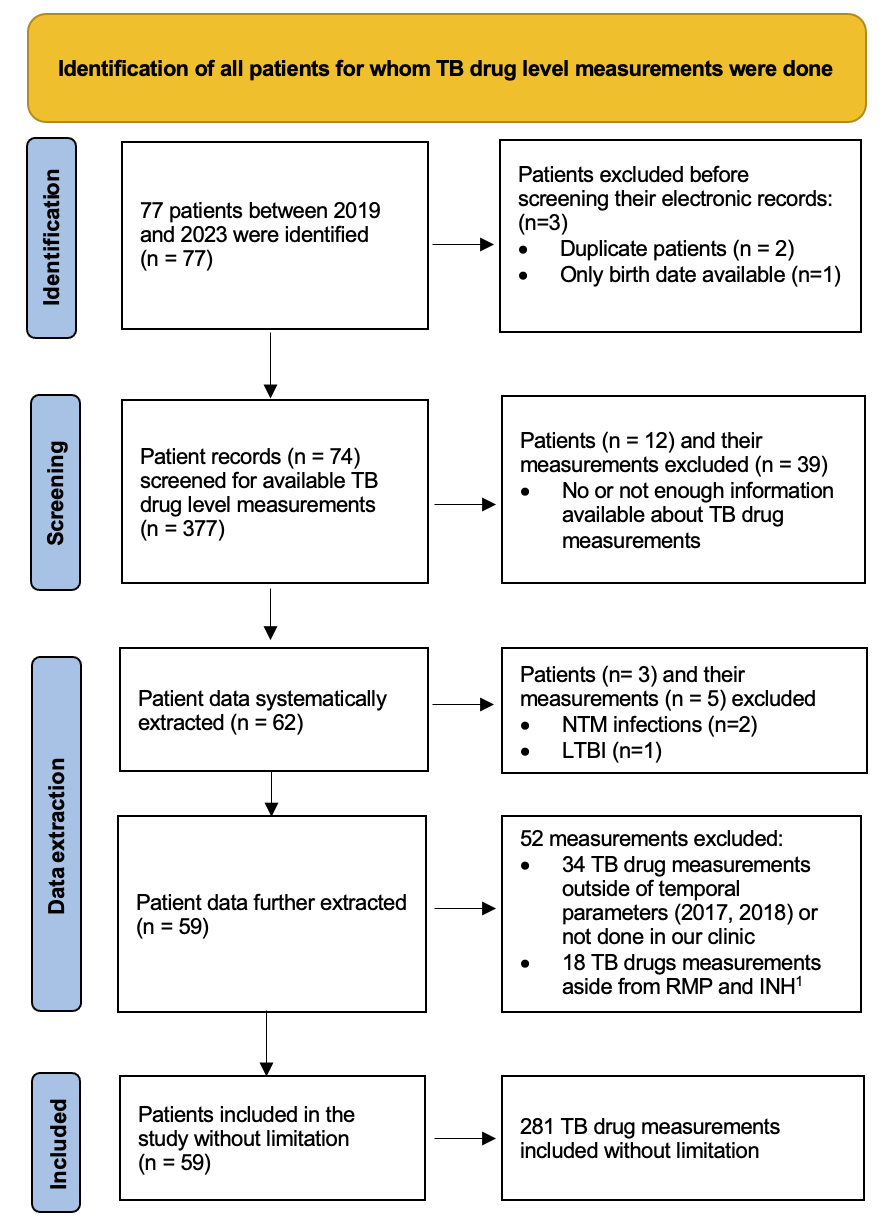


Figure S1: Flow chart of patient and drug level measurement inclusion. TB = tuberculosis; NTM = non-tuberculous mycobacteria; LTBI = latent tuberculosis infection; RMP = rifampicin; INH = isoniazid.


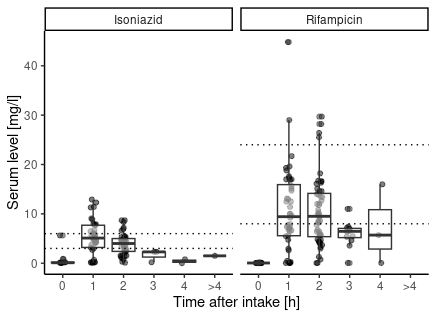


Figure S2: Drug levels for isoniazid and rifampicin at hour 0,1,2,3,4 and >4hours after intake. Dotted lines show target ranges of serum levels respectively.


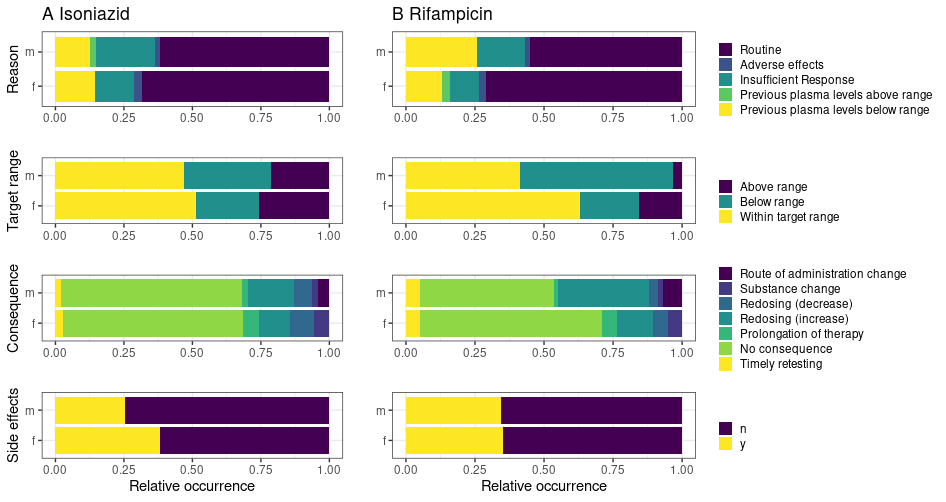
Figure S3: Reasons for drug level testing, target range, consequences of drug level testing and concomitant side effects for isoniazid (A) and rifampicin (B) stratified by gender. f = female; m = male; y = yes; n = no.
